# Remote follow-up based on patient-reported outcomes in patients with chronic kidney disease: A qualitative study of patient perspectives

**DOI:** 10.1371/journal.pone.0281393

**Published:** 2023-02-10

**Authors:** Birgith Engelst Grove, Liv Marit Valen Schougaard, Per Ivarsen, Niels Henrik Hjollund, Annette de Thurah, Caroline Trillingsgaard Mejdahl

**Affiliations:** 1 AmbuFlex, Center for Patient-reported Outcomes, Gødstrup Hospital, Herning, Denmark; 2 Department of Clinical Medicine, Aarhus University, Aarhus, Denmark; 3 Department of Renal Medicine, Aarhus University Hospital, Aarhus N, Denmark; 4 Department of Clinical Epidemiology, Aarhus University Hospital, Aarhus N, Denmark; 5 Department of Rheumatology, Aarhus University Hospital, Aarhus N, Denmark; 6 DEFACTUM—Public Health & Health Services Research, Aarhus, Denmark; University of Warwick, UNITED KINGDOM

## Abstract

**Background:**

Patient-reported outcomes (PROs) are increasingly used in outpatient follow-up. PRO-based remote follow-up offers a new healthcare delivery model, where PROs are used as the basis for outpatient follow-up in patients with chronic kidney disease. However, the patient’s perspective of this novel remote care delivery remains unknown.

**Objectives:**

This study aimed to explore the patients’ experiences using PROs in remote care and how this mode of follow-up may enhance patient engagement.

**Design:**

A qualitative approach was employed, guided by Focused Ethnography and Interpretive Description.

**Participants:**

Purposively, 15 patients with chronic kidney disease experienced with PRO-based remote follow-up in 3 renal outpatient clinics in the Central Denmark Region, were recruited.

**Measures:**

Field studies comprising participant observation in remote PRO consultations and individual, semi-structured interviews with the patients constituted the empirical data. Thematic analysis was performed according to Braun and Clarke’s six-phase process.

**Results:**

PRO-based remote follow-up may enhance patient engagement by a) improving communication, b) increasing disease knowledge, c) inducing flexibility, d) ensuring clinician feedback on PRO data, and e) prompting clinical action. Barriers to enhanced patient engagement were identified as a) lack of feedback on PRO data, b) lower disease knowledge, c) PRO in competition with biomedical data, and d) loss of personal relation.

**Conclusion:**

PRO-based follow-up in remote care holds several advantages for the patients. However, some barriers need clinical awareness before PROs may enhance the patients’ engagement in remote follow-up. Future studies should explore the impact of involving relatives in PRO-based follow-up.

## Introduction

Caring for people with chronic kidney disease (CKD) is complex and demands close monitoring and evaluation of the patients’ symptoms and well-being, as symptoms and comorbidity have a high impact on their daily life [1,2]. Traditionally, patients with CKD have scheduled visits at the hospital [3], and the consultation is mainly focused on biochemical data supported by a dialogue on the patient’s well-being. However, remote monitoring of patients with chronic conditions is rapidly growing, and different healthcare delivery modes occur [4,5]. Initially, patient reported outcomes (PROs) were developed for research with passive registration of the patient’s symptoms. However, PROs are increasingly used actively in routine healthcare delivery to support decision-making and increase patient-centred care for a wide range of chronic conditions [6–8]. PROs allow capturing important disease-specific symptoms and may be a method to create more patient engagement in health care. A PRO measure is “a measurement based on a report that comes directly from the patient about the status of a patient’s health condition without amendment or interpretation of the patient’s response by a clinician or anyone else” [9]. PRO measures are questionnaires that measure patients’ perceptions of the impact of a condition and its treatment on their health [10] Thus, PRO measures are helpful when measuring aspects that are best known to patients or best measured from the patient perspective [11]. The evidence presented thus far supports the idea that PROs in clinical practice potentially increase the patients’ disease knowledge among patients with chronic kidney disease [12,13], improve communication and supportive care [7], and prompt patients to reflect on their health [14–16]. Moreover, PROs may increase the patients’ activation in their follow-up [17], which is consistent with a contribution to strengthening patient engagement [18]. Patient engagement is understood as a process as well as a specific behaviour. It is shaped by the relationship between the patient and provider and the healthcare delivery environment [18]. PROs can enhance patient engagement and activation if clinicians discuss PROs with the patients [17]. Increasing evidence of an association between patient engagement and quality in healthcare is emerging [18,19]. Studies on patients with chronic diseases have shown that engaged patients will have a better understanding of their disease, feel more informed, be more proactive in managing their disease and have better clinical outcomes [20,21].

An ongoing trial in outpatients with chronic kidney disease investigates the possibility of optimising the frequency of hospital consultations using information from remote PROs [22]. When regular outpatient visits are substituted with disease-specific questionnaires, it is termed “PRO-based remote follow-up” and named ‘PRO consultation’ when PROs are used within a consultation. Implementing PROs in a clinical setting encompasses a complex intervention involving the interactions and interpretations of several individuals. Several studies have shown the importance of understanding the potential barriers and facilitators when integrating PROs into a health system [23,24]. Understanding patients’ experiences is essential, particularly when interventions require their active participation [25]. Interpreting the effectiveness of complex interventions is challenging as the intervention may consist of several components that vary but are assumed to contribute to the effect [25]. The patient’s perspective of PRO-based care delivery in remote kidney follow-up remains unknown. Thus, this study aimed to gain insight into how patients perceive the use of PROs in remote outpatient follow-up, with particular attention to how PROs may enhance patient engagement.

## Material and methods

### Study design

A qualitative approach using Interpretive Description (ID) [26] was employed, applying data from individual interviews to capture the patients’ perspectives toward PRO-based remote follow-up. Moreover, participant observations of PRO consultations informed the interview guide, and field notes constituted a supplement to the article’s empirical material using a Focused Ethnographic (FE) approach [27]. The Consolidated Criteria for Reporting Qualitative Research (COREQ) checklist was applied [28].

### Setting

In Denmark, the publicly developed and funded PRO system “AmbuFlex” has been implemented in various hospital departments for improved follow-up in chronic diseases, with patients reporting their symptoms to the clinicians via a web-based platform. The overall aims of AmbuFlex are to enhance the quality of care, increase patient-centred care and reallocate healthcare resources by using PRO measures as the basis for outpatient follow-up [6,29].

In 2019, a multicentre Danish regional randomised controlled trial, the PROKID trial, was established to evaluate whether PRO-based remote follow-up is at least as effective as a usual outpatient follow-up in managing decline in renal function and maintaining patients’ quality of life [22]. Newly referred patients to the outpatient clinics were randomly assigned into using a PRO questionnaire as the basis for remote follow-up.

The questionnaire includes information about blood pressure, weight, self-rated health, renal-specific symptoms and a free-text box [30]. The physicians can access the PRO-based graphical overview embedded in the Electronic Health Record system.

This study is carried out in three renal outpatient clinics at Aarhus University Hospital, Gødstrup Hospital and Viborg Regional Hospital, Central Denmark Region, from September 2021 until March 2022.

### Participant selection and data collection

Data collection was conducted by the first author (BEG). A total of 15 patients enrolled in the PROKID trial, randomised to PRO-based remote follow-up or PRO-based telephone consultation, were contacted and agreed to participate. We decided to use a purposive sample, including patients having the final visit in the PROKID trial, to include patients most familiar with using the PRO-system. We considered the patients’ gender, age and randomisation group to ensure a certain diversity. Semi-structured interviews were continued until enough rich empirical material was generated to inform a robust interpretive description and gain a substantial understanding of patients’ experiences of PRO-based remote follow-up. The patients provided written and verbal consent to participate.

### Participant observation

In total, 15 participant observations were performed during the patients’ final PRO-consultation guided by an observation guide (S1 Table) [31]. The first author was present in the outpatient clinic with the physician, who talked to the patient over the phone. Informal interviews with the physician were conducted, and field notes were taken [32].

### Individual semi-structured interviews

Patients were interviewed shortly after their final PRO consultation to ensure their ability to recall the conversation. Interviews were conducted at the hospital (n = 5), in the patients’ homes (n = 7), and by phone (n = 3) following patients’ wishes (Table 1). An interview guide was developed based on existing literature, discussions within the research team, and data from the participant observations (S2 Table). The interview questions were primarily guided by pre-specified topics related to experiences with PRO-based remote follow-up, symptom monitoring, patient role and communication to identify factors that facilitate or impede engagement in remote follow-up. However, the data from the participant observations and new emerging concepts arising from the ongoing data analysis permitted elaboration on emerging analytic themes.

**Table 1:** Characteristics of the participants.

Characteristics	N	(%)
**Gender**		
Male	9	(60)
Female	6	(40)
**Age**		
50–59	2	(13)
60–69	3	(20)
70–79	7	(47)
80–89	3	(20)
**PRO intervention**		
PRO-based remote follow-up	8	(53)
PRO-based telephone consultation	7	(47)
**Place of interview**		
Patients residence	7	(47)
Hospital	5	(33)
Telephone	3	(20)
**Cohabitation**		
Living with a partner	8	(53)
Living alone	7	(47)
CKD status^a^		
Stage 3b	4	(27)
Stage 4	11	(73)
**Outpatient clinic**		
Aarhus University Hospital	10	(67)
Viborg Regional Hospital	4	(17)
Gødstrup Hospital	1	(16)

Participant information, individual interviews, N = 15.

^a^ CKD = Chronic kidney disease. Stage 3b = estimated glomerular filtration rate 30-44mL/min, stage 4 = estimated glomerular filtration rate 15-29mL/min.

### Data analysis

In accordance with ID, data collection and analysis occurred concurrently, each informing the other in an iterative process [33]. Data analysis was inductively driven and carried out as thematic analysis following Braun and Clarke’s six-phase process for conducting reflexive thematic analysis [34]. 1. Familiarisation with the data, 2. Generating initial codes, 3. Searching for themes, 4. Reviewing potential themes, 5. Defining and naming themes, and 6. Producing the report. The analysis was not a linear process, as we went back and forward through the steps several times. All interviews were audio-recorded and transcribed verbatim. The transcripts were closely read to develop an initial idea of the material. A codebook was developed by BEG and CMT, who evaluated the transcripts line-by-line to identify each potential concept. BEG made the initial coding and grouped the codes into themes and subthemes. Analyses were supported by discussions between BEG and CMT and discussions with co-authors. The data was looked into to support reflexivity, searching for competing conclusions [35]. Data management was supported by the NVivo 12 software program [36].

### Trustworthiness

We have strived to enhance the trustworthiness of the study by ensuring representative credibility, analytic logic, and interpretive authority (ID) [37].

Thus, we used data- and investigator triangulation to enhance the representative credibility.

To ensure the analytic logic of the study, an audit trail was kept; this included information regarding theoretical, methodological, and analytical decisions made throughout the study. Finally, interpretive authority was ensured by documenting the first author’s preconceptions in a reflective journal entailing a critical self-reflection about the researcher’s biases, preferences, and preconceptions.

### Ethical considerations

The Danish Data Protection Agency (ID 1-16-02-260-21) approved the study. An informed written consent form was obtained, and the ethical principles for medical research were followed [38]. According to Danish legislation, this type of study does not require approval by the Biomedical Research Ethics Committee (157/1-10-72-1-21).

## Results

We interviewed 15 newly diagnosed patients with chronic kidney disease stage 3b-5. The patients were predominantly men (60%), with a mean age of 71 years. The characteristics of the participants are shown in Table 1.

For some patients, PRO-based remote follow-up supported their engagement due to improved communication, enhanced disease knowledge and increased flexibility. For some patients, PRO-based remote follow-up did not seem to enhance engagement due to the feeling of being left without support, loss of personal relations and lack of disease knowledge. In the analysis, we identified two main themes: Facilitators and barriers for PRO-based follow-up in remote care to support patient engagement with associated sub-themes (Table 2).

**Table 2:** Facilitators and barriers towards enhancing patient engagement in PRO-based remote follow-up.

Facilitators to patient engagement	Barriers to patient engagement
• PRO improves communication • PRO increases disease knowledge • PRO-based remote follow-up induces flexibility • Clinician feedback increases feelings of security • PRO prompts clinical actions	• Lack of feedback induces a feeling of being unsupported • Self-assessment requires disease knowledge • PRO competes with biomedical data • PRO-based remote follow-up challenges patient-physician relation

The following themes and subthemes are presented and illustrated by participant quotes.

### Facilitators for PRO-based remote follow-up to support patient engagement

#### PRO improves communication

More patients emphasised that PROs provided physicians with an excellent overview of their health status. According to these patients, such an overview led to a more focused communication embracing the whole person, taking mental and social factors into account, rather than just having a biomedical approach. Thereby, these patients stated that PROs facilitated a more holistic assessment. This finding was supported in all the participant observations, in which the PROs were verbally spoken of, and most physicians compared the patient’s health status over time. A free textbox allowed them to elaborate on symptoms or bring new aspects into the conversation, which was very important for some patients. Hence, PROs enabled the patients to take charge of the conversation, which increased the possibility of strengthening patient participation. Several patients argued that PROs constituted their agenda, and completing PROs made them more prepared for the dialogue. As one patient put it:

*’The physician gets to know what he also would have asked if I were sitting in front of him*. *Therefore*, *the questionnaire may even be better because everything is all lined up (*…*) When you are sitting in front of a person*, *sometimes you forget your questions if you haven’t written them down*. *So I think the questionnaire is even better’* (Woman,73 yr)

Thus, PROs helped patients organise their thoughts, identify relevant issues, and express themselves better to the physician during the consultation. Most patients believed that completing PROs informed and enhanced the consultation, making them feel more open and courageous to ask accurate questions. As one patient said:

*’Before*, *I couldn’t think of anything to ask during consultations*. *I’ve probably become more open*. *Before I would think; "you simply can’t ask that kind of question*, *it sounds stupid"*. *Now*, *it’s like I have become more courageous’* (Woman, 72 yr)

Thus, PRO provided patients with a more active role in the communication and strengthening their feelings of being empowered around the decisions made regarding their treatment. With PROs, patients felt the opportunity to initiate an equal dialogue with their physician, allowing them to rethink their health and keep their autonomy rather than leaving the physician responsible for the content. As one patient emphasised:

*’When they ask me something I already have answered in the questionnaire*, *I know a little more about it*, *and at least I have a better understanding of what he’s asking about’* (Woman, 72)

Some patients felt that completing the questionnaire prompted them to discuss certain facets of the disease with the physician. Thus, PROs helped patients talk about topics they might otherwise have avoided. In addition, some patients emphasised that PRO-based remote follow-up improved communication between them and their relatives. Completing PRO with relatives opened up for discussing the patient’s health and well-being and enabled the relatives to play a more active role in the consultation.

#### PRO increases disease knowledge

For most participants, filling in a disease-specific questionnaire caused a deeper reflection on symptoms and awareness of the linkage to the kidney disease. Thus, for some patients, PROs improved their follow-up engagement as it drove them to seek a greater understanding of their chronic condition. Several of the patients stated to be without symptoms. Therefore, illness was only slightly present in these patients’ thoughts during everyday life. For some of these patients, PROs seemed to create a positive focus on the disease and helped them gain more insight and a deeper understanding of the disease. As one patient said:

*’The questions have probably made me think about how my kidneys are doing*, *especially when I cannot feel that there is anything wrong (*..*) For example*, *my tiredness*. *Until they asked me that question*, *I did not know that it had anything to do with my kidneys’* (Woman, 72 yr)

PRO created a room for the patients to take special notice of feeling body signals which increased the possibility of engaging more in managing the disease. Repeated questionnaires made the symptoms more familiar to the patients and made it easier for them to assess their current health status. Filling in the PROs clarified that the severity of the disease was not purely restricted to the level of renal function. One patient reported:

*’Firstly*, *I did not think about it*. *It was not until later that I thought*, *why are they asking me about this*? *Might it have something to do with my kidney function*? *Then I realised what was going on’* (Woman, 72 yr)

Thus, PRO provided another dimension of the picture, clarifying that the patients’ unspecific symptoms were related to kidney disease.

#### PRO-based follow-up induces flexibility

Some patients argued that the main benefit of using PRO remotely was the inherent flexibility of the remote PRO design. The convenience of avoiding transportation back and forward to the hospital and waiting time in the waiting room was important to most patients. One patient expressed:

*’There is not much to talk about when there are no major changes*. *(…)*. *it is a waste of time if I do not have problems*. *So no*, *I have been happy with this (PRO-based remote follow-up)’* (Man, 84 yr)

Using PROs as a part of remote follow-up allowed patients to use the time available and the freedom to choose when to fill in the questionnaire. For some patients, this flexibility increased their feeling of being in charge of their own life and the possibility of actively choosing when and how they wished contact to the healthcare system. As one patient said:

*’It is easier because I have a fairly busy workday*, *so*, *conveniently*, *I can take care of my work and do this (complete the questionnaire) when I get home’* (Man, 60 yr)

The feeling of being more ill when entering the hospital was frequently reported in the interviews, whereas staying at home felt more safe. Some patients compared coming to the hospital with stepping into the role as a guest. They were unfamiliar with the procedures and the unknown culture, making the patients more alert and unsure of their behaviour. As some patients stated, they could talk more openly and freely when talking to the physician over the phone. One patient reported:

*’I am not too fond of the environment at the hospital*. *There are always several people present because someone is in a learning situation*. *You are like a guest*. *It is maybe a little more anonymous in this way (*…*) I think you get to say what you want without holding anything back over the phone*’ (Woman, 73 yr)

Thus, PRO-based remote follow-up helped patients be more relaxed and comfortable with the physicians, which facilitated courage for the patients to engage in the relationship.

#### Clinician feedback increases feelings of security

Several patients were unaffected by the disease in their everyday lives, while others had a high symptom burden. More patients found it very reassuring that the physicians gave them a response to their reports, even when they did not feel the burden of the symptoms asked in the questionnaire. One patient commented:

*’The preparation is on the agenda with the topics I would like to discuss*. *It also lies in the confidence that they will report back to me if there is something that looks a little dangerous*. *So I feel entirely safe’* (Man, 70 yr)

It did not seem to matter to the patients in which form they received feedback—as long as feedback was provided. Contrary, other patients stated that no news was good news. Feeling insecure and distressed due to disease progression seemed to increase the patients’ need for supportive care from the healthcare providers in terms of discussing the issues. As one patient said:

*’As long as it goes well*, *and there are no problems or complications*, *then it can easily work out*, *but when it starts to get serious or something changes*, *I think it is nice to have a face-to-face meeting’* (Woman, 66 yr)

Patients felt like the physician took them seriously, saw beyond the objective biochemical data and engaged with the person behind the numbers when the physician responded to their symptoms. Thus, the patients felt unique and seen and thereby more confident using PRO remotely. More patients emphasised that remote PRO made it safe to stay at home, and they found it very reassuring that the physicians kept an eye on them, even though they did not enter the hospital. One patient commented:

*’Somehow it is reassuring to hear something because my kidney disease can deteriorate without me noticing it’* (Woman, 80 yr)

Thus, feedback enabled patients to feel more confident engaging in remote follow-up.

#### PRO prompts clinical actions

More patients experienced that PROs resulted in actions regarding their treatment. One patient reported:

*’I wrote in the free-text box that I felt something was wrong with my heart*, *so he called the heart department and arranged for me to be seen in their clinic’* (Woman, 73 yr)

Another patient stated that this was the first time anyone had taken notice of his rash, and treatment was initiated. These statements were supported in the participant observations, where several actions were observed, such as a change of medication, extra hospital visits if needed, and contact with home care. All prompted based on the patient’s PRO response. As one patient said:

*’I had reported in the questionnaire that I was dizzy*, *so I was called into the hospital*. *The physician wanted to see me*, *which is something else than talking by phone*. *Here they were able to see how I really felt’* (Man, 84 yr)

When action was done upon the patients’ response, it gave the patients a sense of coherence. Their requests were taken seriously, and they experienced an increased involvement in the decisions taken around their care and, thereby, an increased engagement in their follow-up.

### Barriers for PRO-based remote follow-up to support patient engagement

#### Lack of feedback induces a feeling of being unsupported

Some patients experienced not receiving any feedback on their PRO response from the physicians, which made them feel lost and insecure. One patient said:

*’If you do not get a response*, *you are missing something*. *There are many numbers*, *but they tell me nothing about my health*. *Therefore*, *it is nice to be told that everything looks okay and stable (…) Though I do not always receive a response’* (Woman, 86 yr)

Some patients reported that they did not receive feedback even though they stated they had problems or asked for a contact in the questionnaire. A patient said:

*’There was a time when I didn’t hear anything even though I wanted them to call*. *That made me a bit frustrated’* (Man, 75 yr)

A lack of feedback seemed to leave the patients with a feeling of being unsupported and abandoned. As one patient put it:

*’When you answer these questions*, *and they think something needs to be followed up*, *then you know it happens*. *However*, *if no one is following up on it*, *you wonder and worry even more’* (Man, 70 yr)

Our analysis revealed that when PROs are collected when patients experience low symptom burden, it seemed especially important for them to receive feedback.

#### Self-assessment requires disease knowledge

Most patients argued that the PRO questions were relevant and found it easy to decide which response category to choose. However, some patients had difficulty putting words and numbers on their health status. This issue was supported by a few patients who believed they should be taught how to assess their health. One patient expressed:

*’Then they ask if it has any effect on your health*. *It is difficult to answer when you do not know your disease’* (Woman, 56 yr)

Some patients were concerned about their approach to the questions and had difficulty distinguishing whether they should respond based on their general health, other comorbidities, or take the kidney disease-specific perspective. As one patient said:

*’Some questions have been challenging to answer*. *So how do I respond to that*? *Should my answers be “kidney-related” or “me-related”*? *After all*, *many things come into play*. *So yes*, *it has not been easy*’ (Woman, 56 yr)

This aspect indicated a common topic in the interviews: a lack of basic knowledge about the disease, the specific symptoms and its linkage to the kidney disease. Among some patients, the linkage between the symptoms and kidney disease was known, while others did not reflect upon this. As one patient emphasised:

*’I prefer to visit the doctor out there (*…*) At least for a while*. *I could fill in such a questionnaire from home when I feel more acquainted with the department and more familiar with my disease*. *I probably feel most comfortable and secure if I have an appointment at the hospital*. *Probably*, *it is because I am newly diagnosed–all this is new to me)’* (Man, 75 yr)

Hence, self-assessment seemed to require a basic knowledge of the symptoms.

#### PRO competes with biomedical data

Our analysis displayed that patients had various needs to be taken care of during the consultation. For most patients, talking about PRO was not their prime concern. As one patient said:

*’I have not asked the physician further about my answers to the questionnaire*. *It is more about my blood tests*. *These are the ones I am concerned about and interested in because that is where the real problem lies’* (Woman, 86 yr).

Instead, they emphasised that the most important subject to discuss during the consultations was the level of renal function, stressing that PRO competed with the biomedical data. One patient expressed:

*’After filling out all these papers*, *I am interested in only one thing*. *It is just that the doctor calls up and says that my numbers (blood samples) are acceptable and that we continue as usual’* (Woman, 73 yr)

This downgrading of PRO was also found in the participant observations, revealing that biomedical data was prioritised over PRO in the consultation. Another sign of the physicians’ emphasis on biochemistry was observed in their concluding summaries. In the vast majority of cases, biochemistry was summarised when referring to the patient’s overall health situation.

Moreover, neither the biomedical data nor the PRO-data could stand alone and required a physician to explain and help patients link numbers from the biomedical data and PROs to their current health status. If the patients were unable to link these pieces together, they did not seem to understand the complete picture of their kidney disease, which may contribute to less self-management and level of engagement in care.

#### PRO-based remote follow-up challenges patient-physician relation

Some patients argued that remote PRO consultations often resulted in short, focused conversations with less opportunity for asking additional questions. As one patient emphasised:

’*I prefer face-to-face consultations with the physician rather than at a distance*. *More nuances and opportunities to talk about spontaneous things occur (…)*, *But only because I live close to the hospital*. *If I lived far from the hospital*, *it would be another story*. *Though—the icing on the cake is face-to-face’* (Man, 70 yr)

In addition, some patients felt unprepared for the conversation due to not knowing the exact time for the phone call. According to the patients, PRO-based remote follow-up demanded honesty and openness in talking to a person they did not face. Some patients emphasised this being easy because the conversation could be somewhat on their terms. However, several of the patients stated that remote follow-up required specific knowledge or an initial meeting with the physician for them to open up. As one patient put it:

*’It is not the type of contact that matters*, *just that there is a contact (*…*) I like to know who I’m talking to*, *that it is not a stranger to me that way*. *I have seen and talked to him before (*…*) Gradually you get to know them*, *which I feel comfortable with’* (Woman, 86 yr)

They lacked the sense of the person with whom they were talking. This loss of interpersonal relation made some patients feel insecure and less willing to share personal knowledge. Thus, resulting in reduced patient engagement and thereby less benefit of shared knowledge. Our analysis revealed that the need for a face-to-face appointment was most pronounced when the disease progressed or if they had a serious matter to discuss with the physician.

## Discussion

Our study sought to explore the patients´ perspectives on PROs in remote renal outpatient follow-up. We found signs of PRO-based remote follow-up leading to enhanced patient engagement, but also some barriers seemed to impede patient engagement.

In alignment with several prior studies [15,24,39,40], we found that PROs improved patient-clinician communication. When the physician incorporated the patients’ responses in the dialogue, their understanding of the disease seemed to increase, making it easier for them to handle the burden of kidney disease in everyday life. The collection of PROs alone will not improve patient outcomes unless clinicians, in cooperation with patients, use PROs to guide care [41]. Some of the patients in our study gained an increased knowledge of the disease by completing the disease-specific questionnaire, which was also reported in other studies [42–44]. The patients in the present study were newly diagnosed, as they were all referred to the renal outpatient clinic 1½ years ago. This may explain the lack of knowledge about their kidney disease. Accordingly, our analyses revealed that self-assessment required knowledge specifically related to kidney disease. This notion has not previously been described in relation to PROs but highlights the importance of educating patients to gain a better understanding of their disease. Some patients found it difficult to report their symptoms, some due to a lack of knowledge of the common symptoms of having CKD, and others due to insecurity in assessing their general health. Other studies also confirmed this finding [42,45]. This uncertainty among the patients may lead to frustration in completing PROs and increase patient distress about their ability to manage their condition, which is known to be very important for improving patient engagement in healthcare and interventions [25]. Other studies supported this finding, as they found that a major barrier to the management of chronic kidney disease and engagement in healthcare was a lack of knowledge and confidence in the disease, a passive attitude, and poor patient-physician communication [46,47].

Traditionally, in a renal setting, the assessment of a patient’s level of kidney disease is defined and evaluated via biomedical investigation from laboratory tests [48], while patients’ experience of their disease is incorporated as an add-on to provide information from the patient perspective on the disease impact [41]. Some patients found the communication with the physician to be short and too focused on biomedical data, making it difficult to gain a deeper understanding of their disease. Hence, PRO data were outperformed by the communication around the biomedical data and thus de-emphasised. Experiences from other studies have shown that mainly PRO-data related to clinical data or the treatment tend to be further discussed [42,49]. Observations showed that the physicians primarily emphasised objective measurements such as blood pressure, weight and biochemical measures and thereby may have overlooked the opportunity for PRO data to individualise the communication [18].

Literature describing the implications of the clinicians’ feedback on PROs to the patients is scarce. [50,51]. However, one study described how a lack of feedback might give rise to feelings of rejection and disconnection [15], which was in line with the findings in this study. Providing patients feedback seemed crucial in preventing them from feeling abandoned and insecure about their health status. Moreover, if the patients received feedback, they felt unique and involved and thereby more confident in using PRO remotely. These findings highlight the importance of supporting patients with feedback on their PROs to enhance patient engagement.

Some participants preferred face-to-face consultations, as this represented the patients’ most familiar traditions. Accordingly, patients were more willing to visit the outpatient clinic when their symptoms became more severe. Ladin et al. suggested that telehealth may best supplement rather than supplant in-person patient visits [52]. A recent scoping review recommended developing a hybrid model for long‐term follow‐up care to supplement remote consultations with face‐to‐face consultations [53]. Thus, clinical attention to the increasing need for a face-to-face appointment when the disease progresses or if patients have a serious matter to discuss must be emphasised to increase engagement through personalising healthcare [18]. Telephone consultation has been widely adopted as a safe option for receiving care during the COVID-19 pandemic [54]. However, it was essential for the patients in this study to be familiar with the outpatient clinic and the physician. A prior study aligns with this finding, concluding that patients preferred telephone consultation if they had an already established relationship with the nephrologist [55]. Finally, we found signs that the patient’s relatives were often more involved when the patients completed PROs from their homes, and they often discussed the patient’s health while completing the questionnaire. This finding accords with another study, which showed that telehealth facilitated care partner engagement [52].

Our findings were limited by selecting patients who participated in a remote PRO-based intervention. However, these patients had vast knowledge and expertise on the topic. The patients had moderate to severe kidney disease, thus influencing transferability to the broad population of patients with CKD. However, the findings may be relevant to all populations participating in PRO-based remote follow-up, as the results represented some general perspectives on barriers and facilitators towards using PROs in remote care. Another limitation was that the researcher is the principal investigator in the PROKID trial. According to contemporary theory, the belief of a neutral observer is disputed [56]. To secure reflexivity and reveal the researchers´ preconceptions, a declaration of beliefs before initiation of the study was made [26]. Finally, a few interviews were conducted using the telephone, and the absence of visual cues might have resulted in a loss of non-verbal and contextual data.

A strength of this study was the inductive approach, including observations from the participation study to individualise the interview guide and support the patient*’*s perspectives. Furthermore, we used an established method to increase the transparency of the analysing process [34]. The first and last author did the coding in cooperation, following a process of exploring patterns and relationships in the data.

## Clinical implications

Presumably, remote follow-up for patients with kidney disease will continue to expand, thus requiring outpatient healthcare systems to evolve on existing remote models of care and adapt to the delivery needs of the growing population of older patients with chronic kidney disease [1].

However, it is uncertain how remote follow-up affects care and clinical outcomes compared to outpatient clinic visits, which is further investigated [22].

To enhance the potential of PRO to increase patient engagement and improve the use of PRO-based remote follow-up, we suggest that newly diagnosed patients participate in a kidney school to gain a deeper understanding of their renal disease before being referred to PRO-based remote follow-up. Thus, patients will become more familiar with the outpatient clinic and more confident with their disease and thereby have a greater understanding of the implications of having a chronic condition. This confidence may enable them to engage in their follow-up by giving them the courage to talk about PRO in the consultation. Furthermore, to increase the patient’s level of engagement in care, the physicians must discuss and link PROs with the level of kidney function and convey this linkage as a part of the patient education. This understanding enables the patients to elaborate on their symptoms, thereby providing them with a more active role in the communication. Furthermore, our findings suggest supplementing remote PRO consultations with face‐to‐face consultations when the disease progresses.

To comply with the loss of a personal relationship, we suggest an establishment with a patient responsible physician to be arranged for the individual patient to ensure continuity and confidence so the personal relation consists. We recommend that clinical awareness of providing patients feedback concerning their PROs is essential to prevent them from feeling left without support and insecure about their health status. According to our results, it is not pivotal whether the feedback is given verbal or written, just as long as it is provided. Finally, we suggest that physicians encourage patients to involve their relatives when completing PROs and when participating in remote consultations to support the interaction and understanding of the disease between the patient and their relatives. However, this is an aspect of interest for further investigation, as the association between PROs and the involvement of the relatives was not the primary focus of this study and remains rather unrevealed in the literature.

## Conclusion

Our findings demonstrate a broad variation in the influences of PRO-based follow-up on patient engagement in remote care. Overall, patients had positive perceptions towards PRO-based remote follow-up and appreciated the flexibility and the more profound understanding of the disease gained by discussing PROs with the physician. PROs helped patients identify relevant issues, organise their thoughts and express themselves better to the physician during the consultation, thereby increasing patient engagement. However, some patients had no insight into living with kidney disease and thereby struggled to link PROs with their kidney disease. More patients felt PRO-based remote follow-up resulted in an impersonal relationship with the physician, decreasing their willingness to share important details about their health. Moreover, patients had an increased need for a face-to-face consultation as the disease progressed. Overall, this study suggests some clinical initiatives to enhance the patients’ engagement in remote follow-up.

## Supporting information

S1 Table Observation guide. (DOCX)

S2 Table Interview guide. (DOCX)

S3 Table Preliminary coding and strategy. (PDF)
