# Supplementary material for: Remote follow-up based on patient-reported outcomes in patients with chronic kidney disease: A qualitative study of patient perspectives
Source: PLoS One. 2023 Feb 10;18(2):e0281393. doi: 10.1371/journal.pone.0281393 (PMC9916608; doi:10.1371/journal.pone.0281393)
Supplement: S1 Table — (DOCX) [file pone.0281393.s001.docx]

S1. Table. Observation guide

| **Situation/Focus** | **Notes** |
| --- | --- |
| **Patient:** sex, age, colour code (PRO-based follow-up)  Participants in the consultation  Type of consultation  (PRO-based follow-up or PRO-based telephone consultation) |  |
| **Preparation** | |
| - How does the physician prepare for the consultation? - Time for preparation |  |
| **Assessment of PRO** | |
| - How does the physician assess the PRO response? - Does he notice the colour code? (PRO-based follow-up) - What triggered the red colour code? - Are some symptoms more critical than others? - Does the physician look more into some of the symptoms? - Is the current PRO-response compared to previous answers? |  |
| **Use of data from the Electronic Medical Record (EMR)** | |
| - Biochemistry, medicine, other things? Is the physician familiar with the patient? - How is information from the EMR merged/compared with the PRO responses? |  |
| **Decision-aid (PRO-based follow-up)** | |
| - Contact or no contact with the patient - Does the physician seem to be in doubt/hesitant? - What is the basis for this decision? - Why does the physician choose to contact the patient? PROs, biochemistry, other things? - Why does the physician choose not to contact the patient? |  |
| **Conversation with the patient (telephone)** | |
| - Is it easy to reach the patient by phone? - Who begins the conversation?   - How? - How does the physician frame the conversation?   (I call you because…)   - What does the physician ask the patient? - Is the PRO-response mentioned by the physician or patient? - Does the doctor ask in-depth questions about the patient's PRO response?   - How? - Does the patient ask questions to his response? - Does the discussion of PRO lead to actions? - Has the patient completed the free-text conversation topics? - Besides PRO – which other topics are discussed during the conversation? - Is the patient informed of further decisions? - Who is the most talkative person? Concerning? - Body language? (non-verbal communication and signals) |  |
| **Actions after the consultation** | |
| Handling and documentation   - Documentation in the EMR - Did PROs, biochemistry or other things result in a medical change?   **Supportive care:**  Is the nurse contacted, or is the physician handling things himself?  What actions are performed? E.g. medication adjustments, referral to another department or something else? |  |
