# Supplementary material for: Remote follow-up based on patient-reported outcomes in patients with chronic kidney disease: A qualitative study of patient perspectives
Source: PLoS One. 2023 Feb 10;18(2):e0281393. doi: 10.1371/journal.pone.0281393 (PMC9916608; doi:10.1371/journal.pone.0281393)
Supplement: S2 Table — (DOCX) [file pone.0281393.s002.docx]

S2. Table. Interview guide

| **Topic** | **Question** |
| --- | --- |
| Overall considerations of having been participating in the PROKID trial | - You have participated in the PROKID trial, in which you completed questionnaires before consultations. Overall, I’m interested in how this has been for you? - How has it been to talk to the physician on the phone rather than in person at the hospital? |
| Patient experience of using PRO questionnaires | - How has it been for you to complete questionnaires as part of your outpatient follow-up? - How do you usually fill in the questionnaire (paper, web)? - How much time did you spend completing the questionnaire? - is it OK? Is the time spending acceptable? - Has it been easy or difficult for you to complete the questionnaire? - What do you think about the questions (relevant, challenging to understand)? – did you wonder about some of the questions? - Why? - Do you experience symptoms that the questionnaire doesn’t reflect? - Do you usually have someone to help you fill in the questionnaire? (whom?) - What benefits can you see by answering questionnaires? - What disadvantages can you see in answering questionnaires? - Have you experienced any problems answering questionnaires? (technology, access, etc.?) |
| The patient’s experience in rating health and the need for contact | - How has it been to measure your blood pressure and weight? - Tell me about the first time you had to fill out the questionnaire - what was it like to assess your symptoms? Was it difficult or easy? - How has it been to describe your symptoms? - How has it been to assess the severity of your symptoms? - How has it been to assess your need for contact   (PRO-based follow-up)   - Try to tell me about a time when you were in doubt about whether you needed contact - what made it difficult? (PRO-based follow-up) - How do you determine whether you need the physician to call you or you need a visit at the outpatient clinic? - What do you think about using these questionnaires to leave more responsibility / more decisions to you as a patient |
| The patient’s experience of PRO before and during the consultation | - How did you use the questionnaire to prepare for the consultation? - How do you think the questionnaire has been used in the consultation? - Does the physician ask you about the topics you have written in the questionnaire? - Do you find yourself discussing the topics you would like to discuss? - How do you experience the feedback from the physician? - What kind of feedback do you prefer? - Do you ask in detail for your answers?   - Why/Why not? - Generally, do you and the physician discuss relevant topics during the consultations? - Afterwards, have you often forgotten something you would have brought up? |
| PRO-based follow-up and self-management | Completing the questionnaire:   - Did filling in the questionnaire have any influence on your reflection about your illness - Has the questionnaire had any impact on the actions you take concerning your condition? (e.g. your diet). - Does the questionnaire make you worry about your illness? - Do you find that the questionnaire makes something about having kidney disease easier / more difficult? - Did filling in the questionnaire teach you anything (e.g. about yourself / having a kidney disease)?   Dialogue based on PROs   - If you talked to the physician about your questionnaire responses, did that change your approach to your illness? - Did the conversations about your PRO responses impact your actions concerning your kidney disease? - Did the conversations you have had with the physician teach you anything (e.g. about yourself / having a kidney disease) |
| Relation to the clinic and the physician | - What do you think about replacing a visit to the outpatient clinic with questionnaires? - Did it affect the relationship with the physician/outpatient clinic that you didn’t enter the hospital?   - In which way? - Do you think your conversation with the physician was different (on the phone) comparing with a face-to-face meeting with the physician in the outpatient clinic? - In which way? - If you could choose for yourself, which form for consultation do you prefer? - Could you tell me more about why? |
| Unique questions for each patient, derived from the observation study during the patient’s last PRO consultation | - How do you think your last consultation went? - Was a plan laid out for the next visit? - Individual questions (…) |
| Debriefing | A summary of the topics we have discussed – does the patient think we left relevant questions out? |
