# Supplementary material for: Remote follow-up based on patient-reported outcomes in patients with chronic kidney disease: A qualitative study of patient perspectives
Source: PLoS One. 2023 Feb 10;18(2):e0281393. doi: 10.1371/journal.pone.0281393 (PMC9916608; doi:10.1371/journal.pone.0281393)
Supplement: S3 Table — (PDF) [file pone.0281393.s003.pdf]

Supporting Information, File 3.
Overview of the preliminary NVIVO coding aiming to explore the patients' experiences using PRO-based remote follow-up

**PROKID- patient perspectives. Initial coding of themes**	
**Preliminary themes**	**Descriptions and content in the quotes attached to the theme**
1. What is important to me when I face the healthcare system?	In this theme, patients talk about what is essential to them before, during and after a consultation. It is about the content and the knowledge the patients take with them further in their course. It can also represent thoughts about the consequences of the relationship with the doctor, whether the patient feels seen/heard/recognised, and whether this is important to the patient. In what way does PRO support or counteract what is important to patients?
2. Knowledge on my disease	Patients' perspectives on the impact the disease has on their daily life.
3. PRO-based follow-up	This theme includes quotes about what patients think about their outpatient follow-up. Patients may consider completing the PRO as part of their outpatient follow-up and what it means for them not to have to go to the hospital for a check-up. Do patients feel comfortable answering the PRO rather than the physical interview with the doctor? Patients also reflect on their relationship with the doctor. Here the patients also express their wishes for their future outpatient follow-up
4. Experiences towards completing a PRO questionnaire prior a consultation	Content in this code are quotes where the patient articulates the considerations they have had when answering a questionnaire or considerations about what it means to use a questionnaire as part of an outpatient procedure. Here the patients also talk about how it has been to assess their health
5. Using PRO in the dialogue with the physician	Which experiences do the patients have with the conversations they have had with the physicians during PRO-based follow-up? Is the focus specific in the conversation, and how is PRO verbally spoken of in the dialogue? What experiences have the patients had concerning the response from the physician? How much does PRO take up in the conversation?
6.Self-care/ self-management	Has filling in the questionnaire given rise to other ways of coping with the disease? Has PRO helped to strengthen patients' health-related self-care? There will be quotes that describe the difference/importance the use of PRO (in one way or another) has or does not have for patients' ability/possibility/experience of managing the chronic kidney disease - including managing symptoms, psychological/psychosocial consequences, lifestyle changes etc
7. Feedback	Below this theme, there will be quotes where patients talk about the feedback they have received from their questionnaire responses. What does it mean for the patients whether they received feedback or not? Patients who have commented on this are patients in PRO-based remote follow-up since patients in the PRO-based telephone follow-up are not asked about their need for follow-up
8. PRO and involvement of the relatives	Quotations in this code are about the importance PRO may have for the involvement of the relatives. Maybe the relatives have helped patients fill in the questionnaire, but it may also be about how the patient and the next of kin talk about illness at home - focus on what importance PRO has for relationship/communication with next of kin. Does PRO improve the communication/relationship with the relatives
9. Other	Quotations listed here are considered meaningful but cannot be placed under the above themes. It may, for example, be reasons for taking part in the study or other thoughts into the overall theme

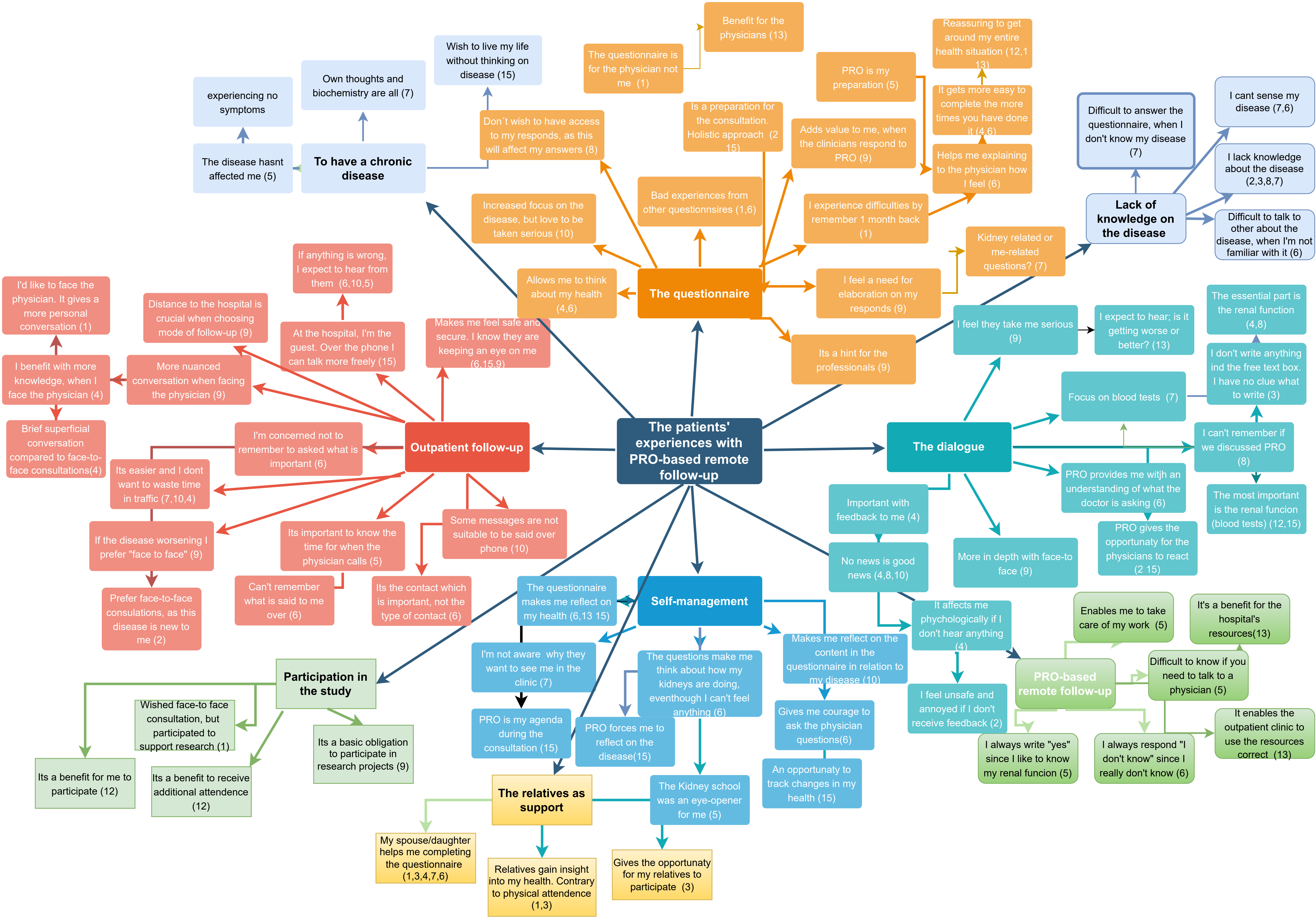
The patients' experiences with PRO-based remote follow-up
To have a chronic disease
The disease hasnt affected me (5)
experiencing no symptoms
Own thoughts and biochemistry are all (7)
Wish to live my life without thinking on disease (15)
Don´t wish to have access to my responds, as this will affect my answers (8)
The questionnaire
The questionnaire is for the physician not me (1)
Benefit for the physicians (13)
Is a preparation for the consultation. Holistic approach (2 15)
PRO is my preparation (5)
Reassuring to get around my entire health situation (12,1 13)
It gets more easy to complete the more times you have done it (4,6)
Helps me explaining to the physician how I feel (6)
Adds value to me, when the clinicians respond to PRO (9)
Bad experiences from other questionnsires (1,6)
Increased focus on the disease, but love to be taken serious (10)
I experience difficulties by remember 1 month back (1)
Kidney related or me-related questions? (7)
Allows me to think about my health (4,6)
I feel a need for elaboration on my responds (9)
Its a hint for the professionals (9)
Lack of knowledge on the disease
Difficult to answer the questionnaire, when I don't know my disease (7)
I cant sense my disease (7,6)
I lack knowledge about the disease (2,3,8,7)
Difficult to talk to other about the disease, when I'm not familiar with it (6)
The dialogue
I feel they take me serious (9)
I expect to hear; is it getting worse or better? (13)
The essential part is the renal function (4,8)
I don't write anything ind the free text box. I have no clue what to write (3)
Focus on blood tests (7)
I can't remember if we discussed PRO (8)
PRO provides me witjh an understanding of what the doctor is asking (6)
The most important is the renal funcion (blood tests) (12,15)
PRO gives the opportunaty for the physicians to react (2 15)
More in depth with face-to face (9)
Important with feedback to me (4)
No news is good news (4,8,10)
It affects me phychologically if I don't hear anything (4)
I feel unsafe and annoyed if I don't receive feedback (2)
PRO-based remote follow-up
Enables me to take care of my work (5)
It's a benefit for the hospital's resources(13)
Difficult to know if you need to talk to a physician (5)
It enables the outpatient clinic to use the resources correct (13)
I always write "yes" since I like to know my renal function (5)
I always respond "I don't know" since I really don't know (6)
Outpatient follow-up
If anything is wrong, I expect to hear from them (6,10,5)
At the hospital, I'm the guest. Over the phone I can talk more freely (15)
Makes me feel safe and secure. I know they are keeping an eye on me (6,15,9)
Distance to the hospital is crucial when choosing mode of follow-up (9)
I'd like to face the physician. It gives a more personal conversation (1)
I benefit with more knowledge, when I face the physician (4)
More nuanced conversation when facing the physician (9)
Brief superficial conversation compared to face-to-face consultations(4)
I'm concerned not to remember to asked what is important (6)
Its easier and I dont want to waste time in traffic (7,10,4)
If the disease worsening I prefer "face to face" (9)
Prefer face-to-face consulations, as this disease is new to me (2)
Its important to know the time for when the physician calls (5)
Can't remember what is said to me over (6)
Some messages are not suitable to be said over phone (10)
Its the contact which is important, not the type of contact (6)
Self-management
The questionnaire makes me reflect on my health (6,13 15)
I'm not aware why they want to see me in the clinic (7)
PRO is my agenda during the consultation (15)
The questions make me think about how my kidneys are doing, eventhough I can't feel anything (6)
PRO forces me to reflect on the disease(15)
Makes me reflect on the content in the questionnaire in relation to my disease (10)
Gives me courage to ask the physician questions(6)
An opportunaty to track changes in my health (15)
The Kidney school was an eye-opener for me (5)
Participation in the study
Wished face-to face consultation, but participated to support research (1)
Its a benefit for me to participate (12)
Its a benefit to receive additional attendence (12)
Its a basic obligation to participate in research projects (9)
The relatives as support
My spouse/daughter helps me completing the questionnaire (1,3,4,7,6)
Relatives gain insight into my health. Contrary to physical attendence (1,3)
Gives the opportunaty for my relatives to participate (3)
